# Clinical Significance of Serum CA125, CA19-9, CA72-4, and Fibrinogen-to-Lymphocyte Ratio in Gastric Cancer With Peritoneal Dissemination

**DOI:** 10.3389/fonc.2019.01159

**Published:** 2019-11-05

**Authors:** Chao Huang, Zitao Liu, Li Xiao, Yongqiang Xia, Jun Huang, Hongliang Luo, Zhen Zong, Zhengming Zhu

**Affiliations:** Department of Gastrointestinal Surgery, The Second Affiliated Hospital of Nanchang University, Nanchang, China

**Keywords:** gastric cancer, peritoneal dissemination, risk factors, decision rules, risk assessment model

## Abstract

**Background:** Relevant serum tumor markers have been indicated to be associated with peritoneal dissemination (PD) of gastric cancer (GC). Fibrinogen has been shown to play an important role in the systemic inflammatory response (SIR) and in tumor progression. However, the clinical significance of the fibrinogen-to-lymphocyte ratio (FLR) in GC with PD has not been studied.

**Methods:** The clinical data of 391 patients with GC were collected, including 86 cases of PD. Then, 1:3 matching was performed by propensity score matching (PSM), and the clinical data of the matched 344 patients were analyzed by univariate and multivariate conditional logistic regression. Classification tree analysis was used to obtain the decision rules and a random forest algorithm to extract the important risk factors of PD in GC. A nomogram model for risk assessment of PD in GC was established by using the rms package of R software.

**Results:** Univariate analysis showed that the factors related to PD in GC were: carbohydrate antigen (CA) 125 (*P* < 0.0001), CA19-9 (*P* < 0.0001), CA72-4 (*P* < 0.0001), FLR (*P* < 0.0001), neutrophil-to-lymphocyte ratio (NLR) (*P* < 0.0001), albumin-to- lymphocyte ratio (ALR) (*P* < 0.0001), platelet-to-lymphocyte ratio (PLR) (*P* = 0.013), and carcinoembryonic antigen (CEA) (*P* = 0.031). Conditional logistic regression found that CA125 (OR: 1.046; *P* < 0.0001), CA19-9 (OR: 1.002; *P* < 0.0001), and FLR (OR: 1.266; *P* = 0.024) were independent risk factors for GC with PD. The accuracy, sensitivity, specificity, positive predictive value and negative predictive value of the decision rules for detecting PD of GC were 89.5, 77.4, 94.0, 82.8, and 91.8%, respectively. According to the important variables identified by the classification tree and random forest algorithm, the risk assessment model of PD in GC was established. The accuracy, sensitivity, and specificity of the model were 91, 89.5, and 79.5%, respectively.

**Conclusion:** CA125 > 17.3 U/ml, CA19-9 > 27.315 U/ml, and FLR > 2.555 were the risk factors for GC with PD. The decision rules and nomogram model constructed by CA125, CA19-9, CA72-4, and FLR can correctly predict the risk of PD in GC.

## Introduction

Gastric cancer (GC) is a malignant tumor with a high incidence ranking fifth among all cancers, but its mortality rate ranks third in the global cancer mortality rate (1). Peritoneal dissemination (PD) is a common distant metastasis mode of GC, and the most important factor leading to shortened survival of patients (2). Relevant guidelines (3, 4) clearly stipulate that GC with PD should not be treated with radical surgery and only with palliative treatment. Therefore, accurate prediction of PD in GC can not only avoid unnecessary laparotomy but also provide the opportunity for early comprehensive treatment such as chemotherapy. At present, computed tomography (CT) examination as a common means to determine the PD of GC has high accuracy and specificity, but the sensitivity is low; PET-CT examination has difficulty in detecting nodules below 5 mm, while the lesions of PD are usually small, so there is a high rate of missed diagnoses (5–7). Although laparoscopic exploration is highly accurate, it is an invasive procedure. Therefore, it is of great significance to identify a non-invasive, highly sensitive and simple prediction method. In recent years, studies have shown that serum tumor markers, especially CA125, have important clinical value in the diagnosis of PD in GC, but the sensitivity of each single indicator was low (8–10). In addition, relevant studies (11, 12) have shown that systemic inflammatory response (SIR) plays an important role in the occurrence, progression and metastasis of tumors. Fibrinogen, a 340-kDa liver glycoprotein, has been proven to play an important role in SIR and tumor progression (13–18). Some studies have also shown that elevated fibrinogen levels are associated with tumor progression and metastasis in advanced gastric cancer (19–21). However, the clinical significance of fibrinogen-to-lymphocyte ratio (FLR) in GC with PD has not been studied. Therefore, the purpose of this study was to investigate the clinical significance of CA125, CA19-9, CA72-4, and FLR in GC with PD.

## Methods

This was a retrospective study and performed according to the guidelines of the Helsinki Declaration. The study was approved by the Ethical Committee of the Second Affiliated Hospital of Nanchang University and written informed consent was obtained from all patients.

### Study Patients

The clinical data of 391 patients with GC were collected, including 86 cases of PD. PD refers to the infiltration of GC tissue into the serosal, and the tumor cells are shed and planted on the peritoneum and organ serosal to form metastatic nodules. The presence of such nodules on CT examination or during surgery was defined as PD group. The absence of such nodules during surgery was defined as without PD group. A total of 391 patients with GC were matched 1:3 according to age, sex, and body mass index (BMI). A total of 344 patients were successfully matched. There were 86 cases in the PD group, including 60 men and 26 women. Their median age was 64 years and their median BMI was 21.38 kg/m^2^. There were 258 cases in the without PD group (diagnosed by laparotomy), including 184 men and 74 women. Their median age was 64 years and their median BMI was 21.51 kg/m^2^. There were no statistically significant differences in age (*P* = 0.900), sex (*P* = 0.773), BMI (*P* = 0.600), or hemoglobin (Hb) (*P* = 0.560) between the two groups (Table 1).

**Table 1:** General characteristics of patients with gastric cancer and comparison of the relevant factors between the two groups (*n* = 344).

**Factor**	**PD** ** (*n* = 86)**	**Without PD** ** (*n* = 258)**	***P* value**
Age (years)	64 (52, 71)	64 (55, 69)	0.900
Sex (*n*)			0.773
Male	60	184	
Female	26	74	
BMI (kg/m^2^)	21.38 (18.86, 22.55)	21.51 (19.48, 23.46)	0.600
Indicators			
NLR	3.58 (2.11, 5.58)	2.44 (1.76, 3.60)	<0.0001
PLR	188.88 (139.74, 266.67)	158.86 (117.68, 211.48)	0.013
ALR	32.13 (24.73, 41.86)	26.15 (22.14, 34.84)	<0.0001
FLR	2.91 (2.16, 4.22)	2.07 (1.52, 2.97)	<0.0001
Hb (g/L)	119.5 (98.50, 133.25)	124.5 (104.75, 139.25)	0.560
CEA (ng/ml)	5.60 (2.89, 17.12)	3.08 (1.86, 5.98)	0.031
CA19-9 (U/ml)	33.60 (13.49, 593.18)	13.73 (7.69, 24.04)	<0.0001
CA125 (U/ml)	41.65 (19.95, 98.23)	8.35 (5.98, 13.85)	<0.0001
CA72-4 (IU/ml)	9.86 (2.18, 23.40)	1.84 (1.20, 7.03)	<0.0001

*PD, peritoneal dissemination; BMI, body mass index; NLR, neutrophil-to-lymphocyte ratio; Hb, hemoglobin; PLR, platelet-to-lymphocyte ratio; ALR, albumin-to- lymphocyte ratio; FLR, fibrinogen-to-lymphocyte ratio*.

### Inclusion and Exclusion Criteria

The inclusion criteria were as follows: (1) Patients with GC were diagnosed by surgery or CT examination; (2) Neoadjuvant radiotherapy or chemotherapy was not administered before surgery; (3) Patients signed informed consent forms; (4) It was approved by the ethics committee of our hospital. The exclusion criteria were as follows: (1) Received surgical treatment within 2 months of enrollment; (2) A history of blood transfusion, bleeding, hemostasis, anticoagulant drugs, or thrombosis; (3) Patient underwent splenectomy; (4) Patients with pregnancy, diabetes, cirrhosis, nephrotic syndrome, acute infection, other distant organ metastasis, or incomplete data were excluded.

### Statistical Analysis

We used the single-sample k-s test to test the normality of the data. If the quantitative data had a normal distribution, they are described as the mean ± standard deviation; otherwise, they are described as the median and interquartile range. Categorical variables are expressed as rate with 95% confidence intervals (CI). Univariate and multivariate logistic regression were used to analyze the indicators of the matched patients. Classification tree analysis was used to obtain the decision rules and a random forest algorithm to extract the important risk factors of PD in GC. A classification tree is a non-linear discriminant method that uses a set of independent variables to gradually decompose a sample into smaller subgroups. This procedure selects the independent variable that has the strongest association with the dependent variable (22). The decision rules provide specific information about risk factors based on rule induction. The direction of individual movement is determined by the answers to the questions on each branch until reaching the end of a terminal node (ellipse) (23). A classification tree model was built by randomly extracting two-thirds of all data (24). The important indicators were ranked by the mean decrease Gini (MDG) involved in the random forest algorithm. The MDG provides a method to quantify which indicator contributes the most to the classification accuracy (25). The threshold of important risk factors was determined by the receiver operating characteristic curve (ROC curve). The optimal cut-off value is commonly used in the “Youden index,” i.e., sensitivity-(1-specificity), where the maximum value of the index is the optimal threshold. The R software rms package was used to construct the nomogram model of risk assessment for PD in GC, and points of various indicators were obtained (26). The points corresponding to the indicators were added to obtain the total points; the higher the total points, the higher the risk of PD of GC. The ROC curve was used to evaluate the accuracy, sensitivity and specificity of the model in predicting PD in GC. A two-sided *P* < 0.05 was considered statistically significant. Data were analyzed using SPSS 22.0 for Windows (SPSS Inc., Chicago, IL, USA) and R (version x64 3.5.1, http://www.r-project.org).

## Results

### Univariate and Multivariate Analysis

**Figure 1:**
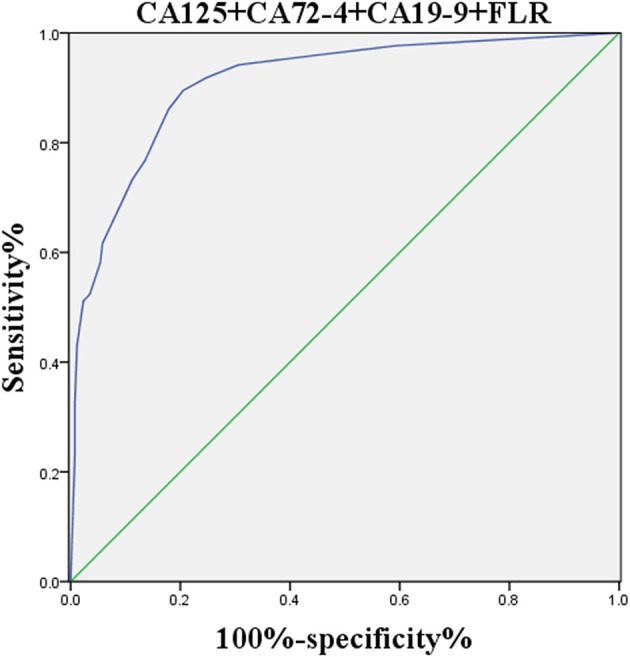
ROC curve of independent risk factors of PD in GC.

Univariate analysis showed that the factors related to PD in GC were as follows: CA125 (*P* < 0.0001), CA199 (*P* < 0.0001), CA72-4 (*P* < 0.0001), FLR (*P* < 0.0001), NLR (*P* < 0.0001), ALR (*P* < 0.0001), PLR (*P* = 0.013), and CEA (*P* = 0.031), and the differences between the two groups were statistically significant (Table 1). Multivariate analysis found that CA125 (OR: 1.046; *P* < 0.0001), CA19-9 (OR: 1.002; *P* < 0.0001), and FLR (OR: 1.266; *P* = 0.024) were independent risk factors for GC with PD (Table 2). The accuracy, sensitivity and specificity of predicting PD in GC were 89.3, 82.6, and 88.4%, respectively (Figure 1).

**Table 2:** Risk factors of PD in GC for multivariate conditional logistic regression analysis.

**Risk factors**	**B**	**SE**	**Wals**	***P* value**	**OR (95% CI)**
CA125	0.045	0.009	26.817	<0.0001	1.046 (1.028, 1.064)
CA19-9	0.002	0.000	13.612	<0.0001	1.002 (1.001, 1.003)
FLR	0.236	0.105	5.063	0.024	1.266 (1.031, 1.555)

### Classification Tree Analysis to Obtain Decision Rules Affecting PD in GC

**Figure 2:**
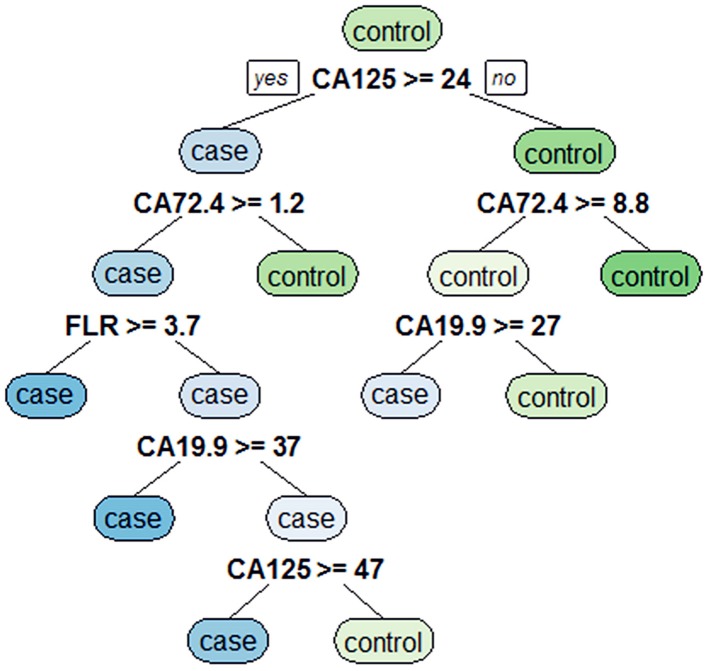
Classification tree for detecting undiagnosed PD in GC.

Eight variables with statistically significant differences were analyzed by using a classification tree to obtain decision rules affecting PD in GC (Figure 2). Four variables were selected through the classification tree program. They were CA125, CA19-9, CA72-4, and FLR. CA125 was the most important determining factor because it was the first-level split of the two initial branches of the decision tree. CA72-4 was the most important determining factor in the second-level split. The accuracy, sensitivity, specificity, positive predictive value and negative predictive value of the decision rules for detecting PD of GC were 89.5, 77.4, 94.0, 82.8, and 91.8%, respectively, indicating that the decision rules had high accuracy and specificity.

### Random Forest Algorithm to Extract Important Risk Factors for PD of GC

**Figure 3:**
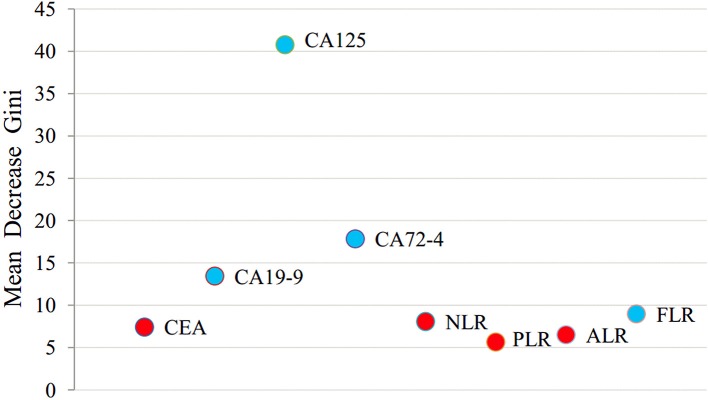
Random forest algorithm for ranking of the importance of all variables.

All variables were analyzed using a random forest algorithm to obtain the importance ranking of the variables (Figure 3). The larger the mean decrease of Gini, the more important the indicator was. The first was CA125, followed by CA72-4, CA19-9, and FLR.

### ROC Curve of Important Risk Factors

**Figure 4:**
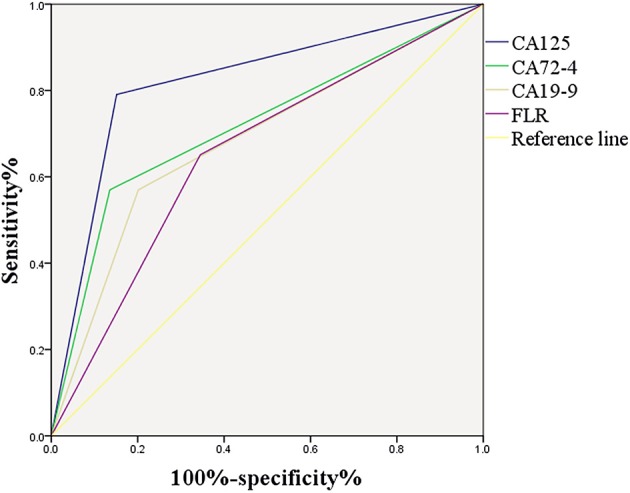
ROC curve of the important risk factors.

Figure 4 shows the correlation between CA125, CA72-4, CA19-9, and FLR with PD in GC. The optimal cut-off values of CA125, CA72-4, CA19-9, and FLR were 17.3 U/ml, 7.25 IU/ml, 27.315 U/ml and 2.555, respectively, according to the ROC curve evaluation. The area under the curve (AUC) of CA125 was 0.820, the 95% CI was 0.764–0.876, the sensitivity was 79.1%, and the specificity was 84.9%. The AUC of CA72-4 was 0.717, the 95% CI was 0.649–0.785, the sensitivity was 57%, and the specificity was 86.4%. The AUC of CA19-9 was 0.684, the 95% CI was 0.615–0.753, the sensitivity was 57%, and the specificity was 79.8%. The AUC of FLR was 0.653, the 95% CI was 0.586–0.720, the sensitivity was 65.1%, and the specificity was 65.5% (Table 3). These data indicate that the accuracy and sensitivity of individual indicators to determine PD were relatively low.

**Table 3:** Relevant results of important risk factors.

**Factors**	**AUC**	**SE**	***P* value**	**95% CI**	**Sensitivity (%)**	**Specificity (%)**	**Cut-off value**
CA125	0.820	0.029	<0.0001	(0.764, 0.876)	79.1	84.9	17.3
CA72-4	0.717	0.035	<0.0001	(0.649, 0.785)	57	86.4	7.25
CA19-9	0.684	0.035	<0.0001	(0.615, 0.753)	57	79.8	27.315
FLR	0.653	0.034	<0.0001	(0.586, 0.720)	65.1	65.5	2.555

### Nomogram Model for Risk Assessment of GC With PD

**Figure 5:**
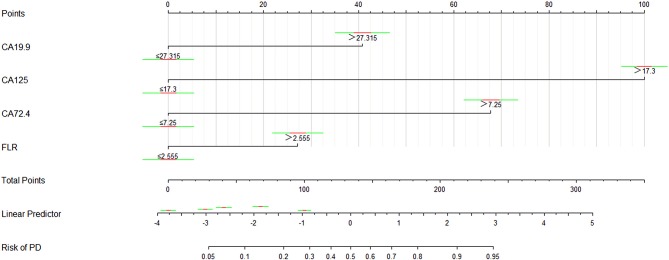
Nomogram of the logistic regression model.

**Figure 6:**
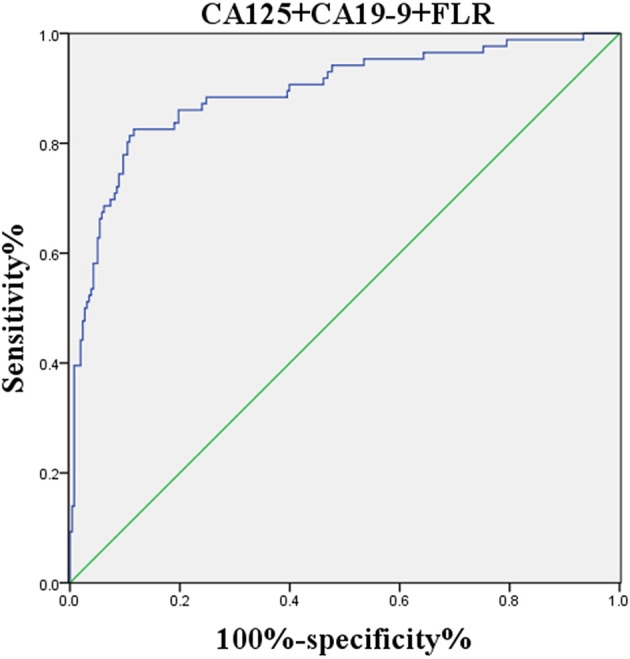
ROC curve of the combined factors.

The logistic regression model of CA125, CA72-4, CA19-9, and FLR was constructed by the R language rms package, and the C statistic of its evaluation was 0.910, demonstrating that the prediction model had high accuracy. Then, the plotting function was constructed, and the nomogram was plotted (Figure 5). A score of CA125 > 17.3 U/ml was 100 points, while a score of CA125 ≤ 17.3 U/ml was 0 points; a score of CA72-4 > 7.24 IU/ml was 68 points, while a score of CA72-4 ≤ 7.24 IU/ml was 0 points; a score of CA19-9 > 27.315 U/ml was 41 points, while a score of CA19-9 ≤ 27.315 U/ml was 0 points; a score of FLR > 2.555 was 27 points, while a score of PAR ≤ 2.555 was 0 points. The total score was 236 points, suggesting that the probability of PD in GC was 91–95%. The risk of GC with PD can be predicted based on the total points (Table 4). The area under the ROC curve of the combined factors was 0.910, the 95% CI was 0.873–0.946, the sensitivity was 89.5%, and the specificity was 79.5%, indicating that the prediction model had high accuracy and sensitivity (Figure 6).

**Table 4:** Relationship between total points and risk of PD in GC.

**Total points**	**Risk of PD (%)**
<30	<5
30–56	5–10
57–85	11–20
86–104	21–30
105–120	31–40
121–134	41–50
135–149	51–60
150–164	61–70
165–183	71–80
184–212	81–90
213–239	91–95
>239	>95

## Discussion

At present, CT examination is a common method to diagnose PD of GC. Although it has high accuracy and specificity, its sensitivity is low. It is also difficult to find nodules < 5 mm in PET/CT examination, and the lesions of PD are usually small, so there is a high rate of missed diagnosis (5–7). Kim et al. (5) found that the sensitivity of CT diagnosis of GC with PD was 28.3–50.9%, and the specificity was 96.2–98.9% through retrospective analysis. Kayaalp et al. (27) analyzed 118 patients with GC using preoperative CT staging, indicating that the sensitivity, specificity, and accuracy of CT in diagnosing PD of GC were 13, 99, and 82%, respectively. In addition, Yajima et al. (28) analyzed 293 patients with advanced gastric cancer, showing that the sensitivity, specificity, and accuracy of CT diagnosis of PD were 50.6, 96.8, and 85.0%, respectively. Laparoscopic exploration is highly accurate, but it is an invasive procedure. In addition, cytological examination of peritoneal lavage during laparotomy can predict PD (29, 30). In this study, 47 out of 86 (54.6%) of patients with negative CT were identified to have PD by laparotomy. SIR plays an important role in the occurrence, progression and metastasis of tumors (11, 12). Chronic inflammation produces a microenvironment that facilitates tumor growth and metastatic spread of tumor cells (31, 32). Lymphocytes are major anticancer factors. As an important part of tumor-specific immune responses, they play a specific role in killing tumor cells by inducing cytotoxic cell death and cytokine production to mediate host immune responses, thereby inhibiting tumor cell proliferation (12, 33). Eo et al. (34) found that preoperative lymphocytosis was an independent risk factor for the prognosis of GC, and the overall survival and disease-free survival rates of patients with preoperative lymphocytosis were lower. In addition, Arigami et al. (35) showed that the density of the cluster of differentiation (CD) 3+ tumor-infiltrating lymphocytes in patients with GC decreases during tumor progression.

Fibrinogen plays an important role in blood coagulation, cell-cell adhesion, and the inflammatory response (19). Meanwhile, the coagulation cascade plays an important role in tumor progression and metastasis (36). Fibrinogen, as an important component of the coagulation cascade, has been proven to be an important regulator of SIR and tumor progression (13–18). Fibrinogen provides a stable framework for the tumor extracellular matrix, thereby promoting tumor angiogenesis and enhancing adhesion, migration, and invasion of tumor cells (37); in addition, fibrinogen, as an acute phase protein, may induce tumor cell proliferation and invasion by mediating the initial adhesion of leukocytes to endothelial cells, thus promoting the release of pro-inflammatory cytokines (38). Previous studies (39) have reported that platelets and fibrinogen interact to protect tumor cells from the phagocytosis of natural killer cells. It is well-known that elevated fibrinogen is a predictor of cardiovascular events and mortality from chronic kidney disease (40). In addition, recent studies have shown that elevated fibrinogen is involved in the malignant behavior of many types of cancer (41–43) and it promotes cancer cell growth, invasion, and metastasis (16–18). Relevant studies have also shown that elevated fibrinogen is associated with tumor progression and metastasis in advanced gastric cancer (19–21). Kijima et al. (44) found that fibrinogen not only plays an important role in the occurrence and metastasis of tumors but can also be used as an observation indicator for the prognosis of esophageal squamous cell carcinoma. Suzuki et al. (45) demonstrated that preoperative plasma fibrinogen elevation was associated with tumor progression and poor overall survival in patients with GC. In addition, Palumbo et al. (46) indicated that in animal experiments fibrinogen deficiency can significantly reduce the potential of tumor metastasis. The current study found that the FLR of the GC with PD group was higher than that of the without PD group and the FLR was an independent risk factor for PD in GC. In addition, FLR was also selected in the model of the classification tree. Meanwhile, when the random forest algorithm was applied to extract the important risk factors for PD of GC, these results also showed that FLR was an important risk factor.

CA125 is a tumor-associated glycoprotein antigen and an increase in its serum level is associated with many malignant tumors, and an increased level of CA125 in peritoneal lavage is associated with PD and a poor prognosis of GC (47). Nakata et al. (48) first analyzed the clinical data of 384 patients with GC and concluded that the accuracy, sensitivity, and specificity of serum CA125 in the diagnosis of PD of GC were 90.8, 39.4, and 95.7%, respectively. Hwang et al. (49) showed that the accuracy, sensitivity and specificity of serum CA125 in the diagnosis of PD in GC were 91.5, 38.6, and 98.4%, respectively. Emoto et al. (8) indicated that serum CA125 was significantly positively correlated with GC with PD, and its diagnostic sensitivity was 46%. In addition, Fujimura et al. (10) found that the accuracy, sensitivity, and specificity of serum CA125 in the diagnosis of PD of GC were 76, 55, and 100%, respectively (14). This study found that serum CA125 of the GC with PD group was higher than that of the without PD group and serum CA125 was an independent risk factor for PD in GC. The accuracy, sensitivity, and specificity of serum CA125 in the diagnosis of PD in GC were 82, 79.1, and 84.9%, respectively. In addition, serum CA125 was the most important determining factor in the model of the classification tree. Meanwhile, when the random forest algorithm was applied to extract the important risk factors of GC with PD, the results also showed that serum CA125 was the most important risk factor.

CA72-4 is a tumor-associated glycoprotein that can be found in a variety of cancers, and has a high specificity for the diagnosis of GC (50). Ca19-9 is a kind of glycoprotein that exists in the form of mucin in serum. Its level *in vivo* is affected by the size, metastasis, and invasion of tumors, and it is mainly used for the diagnosis of pancreatic cancer, GC, and colon cancer (51). Shimada et al. (52) found through systematic review that serum CA72-4 and CA19-9 were of great value in monitoring the recurrence and metastasis of patients with GC. Emoto et al. (8) showed that the sensitivity of serum CA72-4 and CA19-9 in the diagnosis of GC with PD were 36 and 45%, respectively, and that serum CA72-4 was a useful indicator of prognosis. Hamazoe et al. (53) demonstrated that the positive rate of serum CA72-4 in patients with PD of GC was significantly higher than that of CEA. Ucar et al. (54) reported that serum levels of CA72-4 and CA19-9 were significantly increased in patients with PD of GC; high preoperative serum CA72-4 levels in patients with GC were associated with a higher risk of death. In addition, Hackbarth et al. (9) indicated that GC with PD can cause peritoneal inflammation, leading to a significant increase in CA19-9 levels, so CA19-9 can be considered as an indicator for the diagnosis of PD in GC. This study found that serum CA72-4 and CA19-9 of the GC with PD group was higher than that of the without PD group and serum CA19-9 was an independent risk factor for GC with PD. The accuracies of serum CA72-4 and CA19-9 in the diagnosis of PD in GC were 71.7 and 68.4%, respectively; the sensitivities were 57 and 57%, respectively; and the specificities were 86.4 and 79.8%, respectively. In addition, serum CA72-4 is the second most important determining factor in the model of the classification tree. Meanwhile, when the random forest algorithm was applied to extract the important risk factors of GC with PD, those results also showed that serum CA2-4 and CA19-9 were both relatively important risk factors.

In addition, the decision rules involved four variables (CA125, CA19-9, CA72-4, and FLR) selected by the classification tree program. The accuracy, sensitivity, specificity, positive predictive value and negative predictive value of the decision rules for detecting PD of GC were 89.5, 77.4, 94.0, 82.8, and 91.8%, respectively. Meanwhile, we combined serum CA125, CA19-9, CA72-4, and FLR to construct a nomogram model for risk assessment of GC with PD, and showed that the accuracy, sensitivity and specificity were 91, 89.5, and 79.5%, respectively.

This study has some limitations. First, this is a single-center retrospective study, so there may be some bias. Second, the sample size is not sufficiently large. Third, several other inflammatory markers related to tumor progression, such as interleukin-6, C-reactive protein, and hypersensitive C-reactive protein, were not included in this study. Therefore, multicenter large-scale prospective randomized controlled trials are necessary.

## Conclusion

This is the first study to apply FLR to predict PD of GC and to establish decision rules and risk assessment models for PD of GC. We found that CA125 > 17.3 U/ml, CA19-9 > 27.315 U/ml, and FLR > 2.555 were risk factors for GC with PD; the decision rules and nomogram models constructed by CA125, CA19-9, CA72-4, and FLR can correctly predict the risk of GC with PD, providing certain guidance for preoperative diagnosis of PD in GC.

## Data Availability Statement

The datasets generated for this study are available on request to the corresponding author.

## Ethics Statement

All procedures were performed in accordance with the guidelines of the Helsinki Declaration. The study was approved by the Ethical Committee of the Second Affiliated Hospital of Nanchang University and written informed consent was obtained from all patients.

## Author Contributions

CH and ZZh designed the study and wrote the manuscript with contribution from all authors. CH, ZL, LX, and YX collected clinical data. CH and HL analyzed the data. ZZo and JH provided critical comments for this paper. All authors read and approved the final version of the paper.

### Conflict of Interest

The authors declare that the research was conducted in the absence of any commercial or financial relationships that could be construed as a potential conflict of interest.
